# Improving Photosynthetic Capacity, Alleviating Photosynthetic Inhibition and Oxidative Stress Under Low Temperature Stress With Exogenous Hydrogen Sulfide in Blueberry Seedlings

**DOI:** 10.3389/fpls.2020.00108

**Published:** 2020-02-28

**Authors:** Xuedong Tang, Baiyi An, Dongmo Cao, Ru Xu, Siyu Wang, Zhidong Zhang, Xiaojia Liu, Xiaogang Sun

**Affiliations:** College of Horticulture, Jilin Agricultural University, Changchun, China

**Keywords:** hydrogen sulfide, low temperature, blueberry seedlings, photosynthetic, reactive oxygen species, proline

## Abstract

In this study, we investigated the mechanism of photosynthesis and physiological function of blueberry leaves under low temperature stress (4–6°C) by exogenous hydrogen sulfide (H_2_S) by spraying leaves with 0.5 mmol·L^–1^ NaHS (H_2_S donor) and 200 μmol·L^–1^ hypotaurine (Hypotaurine, H_2_S scavenger). The results showed that chlorophyll and carotenoid content in blueberry leaves decreased under low temperature stress, and the photochemical activities of photosystem II (PSII) and photosystem I (PSI) were also inhibited. Low temperature stress can reduce photosynthetic carbon assimilation capacity by inhibiting stomatal conductance (*G*_s_) of blueberry leaves, and non-stomatal factors also play a limiting role at the 5^th^ day of low temperature stress. Low temperature stress leads to the accumulation of Pro and H_2_O_2_ in blueberry leaves and increases membrane peroxidation. Spraying leaves with NaHS, a donor of exogenous H_2_S, could alleviate the degradation of chlorophyll and carotenoids in blueberry leaves caused by low temperature and reduce the photoinhibition of PSII and PSI. The main reason for the enhancement of photochemical activity of PSII was that exogenous H_2_S promoted the electron transfer from *Q*_A_ to *Q*_B_ on PSII acceptor side under low temperature stress. In addition, it promoted the accumulation of osmotic regulator proline under low temperature stress and significantly alleviated membrane peroxidation. H_2_S scavengers (Hypotaurine) aggravated photoinhibition and the degree of oxidative damage under low temperature stress. Improving photosynthetic capacity as well as alleviating photosynthetic inhibition and oxidative stress with exogenous H_2_S is possible in blueberry seedlings under low temperature stress.

## Introduction

Hydrogen sulfide H_2_S has dual effects on plant growth and development. A high concentration of H_2_S can cause cytotoxicity, while a low concentration of H_2_S does not cause toxicity to plants and may act as a signaling molecule (Duan et al., 2015; Li et al., 2016). Recently, many studies have found that H_2_S can regulate plant growth and development, such as inducing plant seed germination (Liu and Lal, 2015), improving photosynthetic capacity (Coyne and Bingham, 1978; Chen et al., 2011), regulating stomatal movement (Lisjak et al., 2011; Scuffi et al., 2014), promoting the development of lateral roots (Jia et al., 2015; Fang T. et al., 2014), regulating secondary metabolism of sugar, polyamines, organic acids and amino acids (Shi et al., 2015; Chen et al., 2016), participating in protein modification (Mustafa et al., 2009), maintaining ion balance in plants (Wang et al., 2012; Lai et al., 2014), delaying ripening and senescence of postharvest fruits during storage (Fu et al., 2014; Hu et al., 2014), and improving antioxidant capacity (Luo et al., 2015). In addition, H_2_S has been shown to participate in the regulation of resistance (Hua et al., 2010; Christou, 2013; Jin et al., 2018). The application of exogenous H_2_S could promote plant growth and seed germination (Zhang et al., 2010a), increase the survival and regeneration ability of *Nicotiana tabacum* cells under heat stress, alleviate cell electrolyte leakage and malondialdehyde (MDA) accumulation after heat shock (Li et al., 2012), and alleviate the inhibition of heavy metal stress on plant root growth (Chen et al., 2013). H_2_S also interacts with other hormones and signaling substances in plants (Hancock and Whiteman, 2016). H_2_S can alleviate the inhibition of salt stress on the growth of *Medicago sativa* seedlings and is closely related to the increase of NO content (Wang et al., 2012). Under lead stress, H_2_S and NO can improve the antioxidant system and mineral balance of sesame by interacting (Amooaghaie et al., 2017). H_2_S can be used as upstream signaling molecule of H_2_O_2_ to promote mung bean (*Vigna radiata*) seed germination (Li and He, 2015). H_2_S can be used as a signal molecule of salicylic acid (SA) to participate in Cd tolerance in *Arabidopsis thaliana* (Qiao et al., 2015). H_2_S has a complex relationship with Ca^2+^ in regulating abiotic stressors such as high temperature (Li et al., 2012), Cr^6+^ (Fang H. et al., 2014), and drought (Jin et al., 2013). Cheng et al. (2013). found that H_2_S could inhibit the production of reactive oxygen species and ethylene and alleviate the death of *Pisum sativum* root tip induced by hypoxia by simulating flooding and a hypoxic environment.

Low temperature is one of the most common adversities facing agricultural production in cold regions. Low temperature stress inhibits plant growth and physiological function, which is also related to the decrease of photosystem II (PSII) and photosystem I (PSI) activity (Shen et al., 1990), the limitation of assimilation synthesis (Strauss et al., 2010), the decrease of dark reaction-related enzymes activity, and the disturbance of active oxygen metabolism (Joanna et al., 2019). In the early spring in northern China, blueberries often suffer from low temperature damage, so improving low temperature tolerance is of great significance in the flowering and fruiting stages of blueberries. Sodium hydrosulfide (NaHS) can form H_2_S in solution, and hypotaurine (Hypotaurine) can scavenge H_2_S by directly binding with sulfides. Although a large number of studies have proved that exogenous NaHS with appropriate concentration can improve plant resistance to abiotic stresses, there are few studies on H_2_S improving plant resistance to low temperature, especially on photosynthetic function of blueberry under low temperature stress.Therefore, NaHS and Hypotaurine are often used as the donor and scavenger of H_2_S, respectively (Wang et al., 2012). In this paper, the effects of exogenous NaHS and Hypotaurine on the photosynthetic function and physiological characteristics of blueberry leaves under simulated low temperature stress were studied. The aim of the study was to explore the mechanism of exogenous H_2_S regulating the physiological characteristics and photosynthetic function of blueberry leaves under low temperature stress and to provide theoretical basis for improving the low temperature tolerance of blueberry seedlings in the greenhouse and during transplanting.

## Materials and Methods

### Materials and Treatments

This study was conducted using annual seedlings of Meiden, a lowbush blueberry cultivar with strong cold resistance, which is popular in northern China, at the College of Horticulture, Jilin Agricultural University, Jilin, China in 2018. The seedlings were seeded in pots with a top diameter of 20 cm, a bottom diameter of 16 cm, and a height of 20 cm. The pots were filled with well mixed turf soil and vermiculite (volume ratio 2:1). Plants were grown in an artificial climate chamber with a temperature of 25°C, a light intensity of 400 μmol·m^–2^·s^–1^, and a light cycle of 12 h light, 12 h dark.

Thirty seedlings with similar growth were selected for the experiment. The treatment group was sprayed with 0.5 mmol·L^–1^ NaHS and 200 μmol·L^–1^ Hypotaurine, respectively, and the control group was treated with distilled water. The leaves were sprayed uniformly on both sides until the solution on the leaves formed fine mist-like droplets. Each treatment contained 10 plants as repeats. After spraying NaHS and Hypotaurine, the droplets on the leaf surface were allowed to dry naturally. After three days, all groups were removed to the temperature controlled growth cabinet and the cabinet was maintained at 4–6°C. Light intensity and humidity were identical for all treatments. Physiological indexes were determined before the treatment (marked as day 0) and at the 2^nd^ and 5^th^ days after the treatment.

### Parameters and Methods of Determination

#### Determination of Fast Chlorophyll Fluorescence Induction Curve (OJIP) and 820 nm Light Reflection Curve

The unfolded penultimate leaves of blueberry in different treatments were selected and dark adapted for 30 min by dark adaptation clips. The OJIP curves and 820 nm light reflection curves of leaves after dark adaptation were measured using a Hansatech M-PEA (Multi-Function Plant Efficiency Analyser). Five repetitions were carried out for each treatment (biological experiments). According to the formulas *V*_O–P_=(*F*_t_–*F*_o_)/(*F*_P_–*F*_o_) and *V*_O–J_=(*F*_t_–*F*_o_)/(*F*_J_–*F*_o_), OJIP curves were standardized by O–P and O–J to obtain *V*_O–P_ and *V*_O–J_ curves. The relative variable fluorescence *V*_J_ of J point (2 ms) on *V*_O–P_ curve and the relative variable fluorescence *V*_K_ of K point (0.3 ms) on the *V*_O–J_ curve were also defined. In the formula, *F*_t_ is the relative fluorescence intensity at each time point on the OJIP curve, while *F*_o_*, F*_J_ and *F*_P_ represent the relative fluorescence intensity at 0.01, 2, and 1,000 ms time points, respectively. The standard *V*_O–P_ and *V*_O–J_ curves of blueberry leaves in different treatments were compared with those of CK curves and expressed as △*V*_O–P_ and △*V*_O–J_. A JIP-test analysis was conducted on the OJIP curve to obtain the maximum photochemical efficiency of PSII (*F*_v_/*F*_m_), the performance index of PSII based on absorption (*PI*_ABS_), and the JIP-test analysis of OJIP curves following the method described by Strasser et al. (1995). The activity of the PSI reaction center is reflected by the relative decrease (△*I*/*I*_o_) of the 820 nm light reflection curve (MR820 nm) signal and the slope of the MR820 nm curve as it descends in the initial stage (1–2.5 ms). *I*_o_ and △*I* represent the maximum of the reflected signal and the difference between the maximum and minimum reflected signals in the 820 nm light reflection curve, respectively (Zhang et al., 2018b).

#### Determination of Photosynthetic Gas Exchange Parameters and Carboxylation Efficiency (*CE*)

The unfolded penultimate leaves of blueberry in different treatments were selected to measure the photosynthetic gas exchange parameters by Li-6800 photosynthetic system (Licor Corporation, UK). The net photosynthetic rate (*P*_n_), stomatal conductance (*G*_s_), transpiration rate (*T*_r_), and intercellular CO_2_ concentration (*C*_i_) of blueberry leaves in different treatments were measured under the conditions of 400 μmol·mol^–1^ CO_2_ fixed by CO_2_ cylinder and 1,000 μmol·m^–2^·s^–1^ PFD set by built-in light source. The measurements were repeated five times (biological experiments). The light intensity PFD was fixed to 1,500 μmol·m^–2^·s^–1^ (saturated light intensity) using the built-in light source of the Li-6400 photosynthetic system. The CO_2_ concentration (Ci) was controlled by CO_2_ cylinders to 400, 300, 200, 150, 100, and 50 μmol·mol^–1^, respectively, to obtain the corresponding *P*_n_. The initial slope of *P*_n_–*C*i response curve was considered the carboxylation efficiency (CE).

#### Determination of Physiological Indexes

The content of chlorophyll a (Chla), chlorophyll b (Chlb), and carotenoids (Car) was determined by visible spectrophotometry with 80% acetone extraction (Lichtenthaler, 1987). The proline (Pro) content was measured by acidic ninhydrin colorimetry with 3% sulfosalicylic acid boiling water extraction (Bates et al., 1973). The measurement of H_2_O_2_ content followed the methods described by Alexieva et al. (2001). To monitor lipid peroxidation and membrane integrity, malondialdehyde (MDA) concentration was determined with fresh leaves as described previously (Wang et al., 2010). All physiological indexes were repeated three times (biological experiments).

### Statistical Analysis

Excel (2003) and SPSS (22.0) software were used for statistical analysis. All data were the means ± standard error (SE). One-way ANOVA and least significant difference (LSD) were used for the comparison of the differences between different datasets. A *P* value less than 0.05 was considered statistically significant.

## Results and Analysis

### Chlorophyll and Carotenoid Content

**Figure 1:**
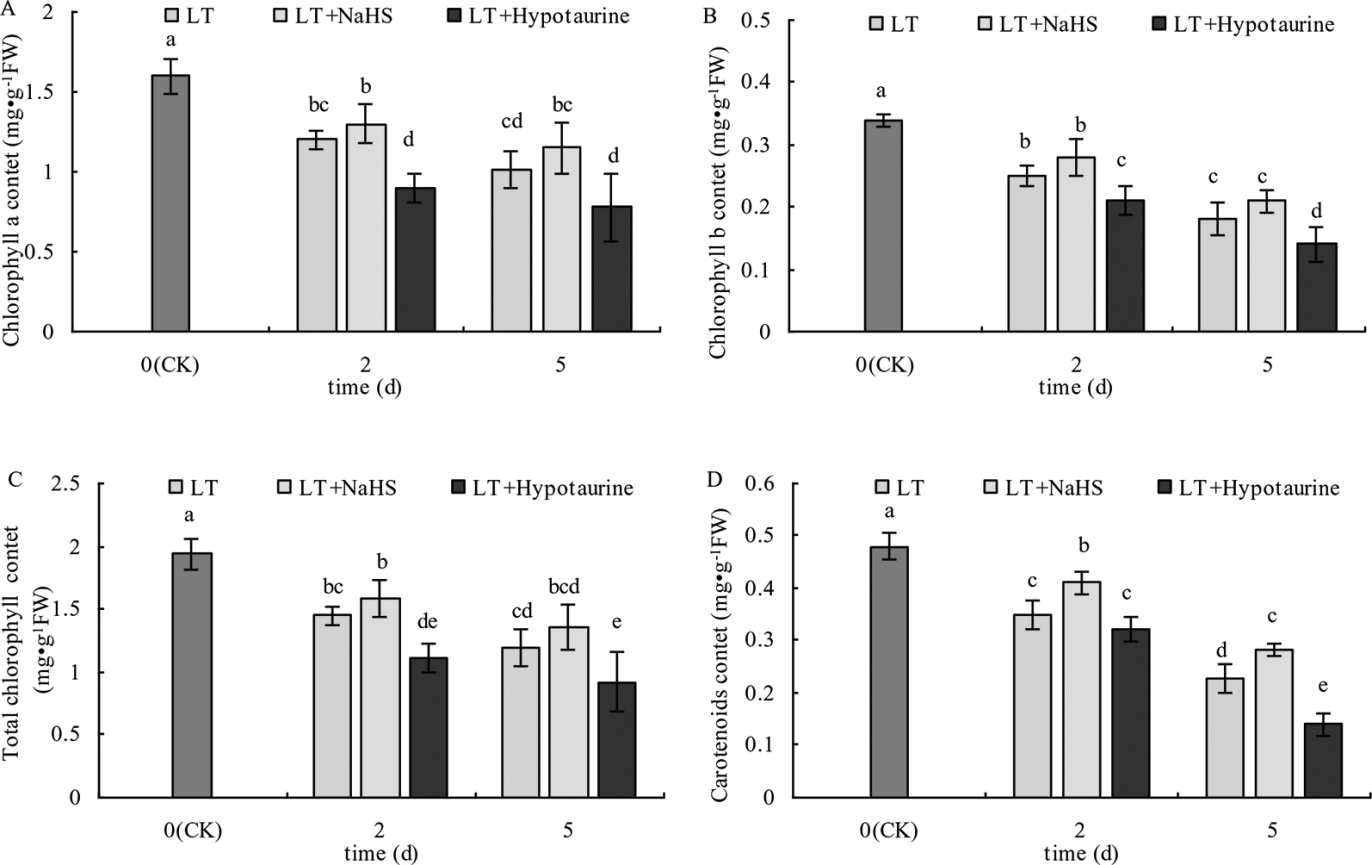
Effects of exogenous NaHS and Hypotaurine on chlorophyll a **(A)**, chlorophyll b **(B)**, total chlorophyll a **(C)**, and carotenoid **(D)** contents in blueberry leaves under low temperature stress. The data in the figure are from three replicated experiments (n = 3), and represent means ± standard error (SE). Different small letters show significant differences (*P* < 0.05). CK: room temperature control at 25°C; LT: low temperature treatment; LT + NaHS: low temperature treatment at 4–6°C after spraying 0.5 mmol·L^–1^ NaHS; LT + Hypotaurine: low temperature treatment at 4-6°C after spraying leaves with 200 μmol·L^–1^ hypotaurine.

As shown in **Figure 1**, the Chl a, Chl b, Chl (a+b), and Car content in blueberry leaves decreased significantly under low temperature stress, and the extent of the reduction increased with the increased duration of low temperature stress. The Chl a, Chl b, and Chl (a+b) content in blueberry leaves treated with NaHS under low temperature stress increased to varying degrees compared with those treated with LT, but the difference was not significant (*P* > 0.05). The Chl a, Chl b, and Chl content after treatment with Hypotaurine was significantly lower than that of the LT treatment (*P* < 0.05). Car content of blueberry leaves treated with NaHS was 17.14% (*P* < 0.05) and 23.25% (*P* < 0.05) higher than the leaves treated with LT at the 2^nd^ and 5^th^ day of low temperature, respectively. In contrast, the Car content in blueberry treated with Hypotaurine was 8.29% (*P* > 0.05) and 38.59% (*P* < 0.05) lower than that in the LT treatment, respectively.

### OJIP Curve and Photochemical Efficiency of PSII

**Figure 2:**
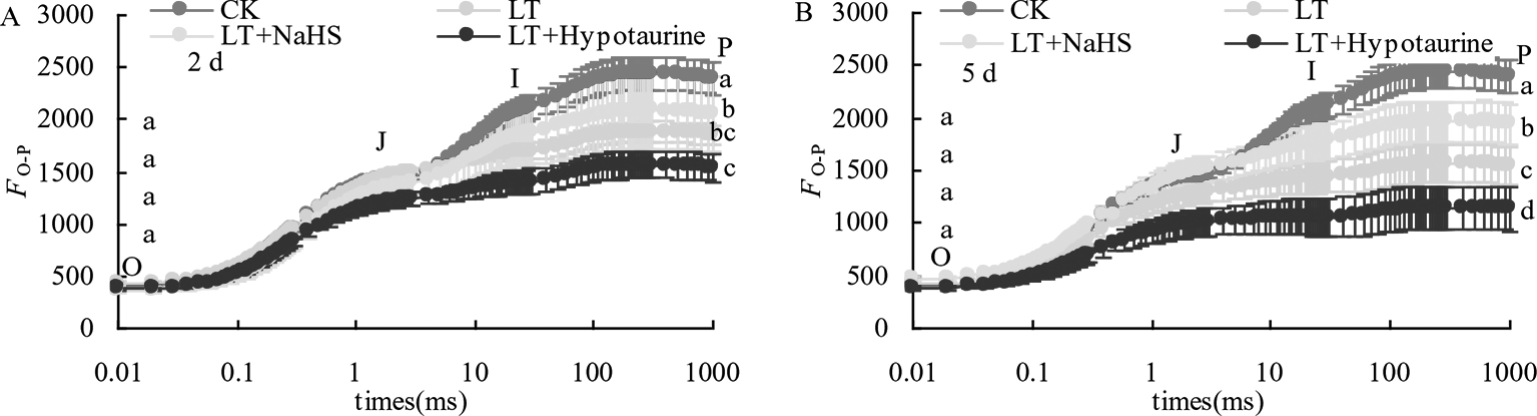
Effects of exogenous NaHS and Hypotaurine on OJIP curves of blueberry leaves under low temperature stress at the 2^nd^ **(A)** and 5^th^ **(B)** day. The data in the figure are from five replicated experiments (n = 5). Different small letters show significant differences (*P* < 0.05). CK: room temperature control at 25°C; LT: low temperature treatment; LT + NaHS: low temperature treatment at 4–6°C after spraying 0.5 mmol·L^–1^ NaHS; LT + Hypotaurine: low temperature treatment at 4-6°C after spraying leaves with 200 μmol·L^–1^ hypotaurine.

The results in **Figure 2** showed that low temperature stress significantly changed the OJIP curve of blueberry leaves. The relative fluorescence intensity *F*_o_ of point O changed little, whereas the *F*_p_ of point P decreased significantly, and the variation at the 5^th^ day of low temperature treatment was significantly larger than that at the 2^nd^ day. NaHS treatment significantly alleviated the decrease of *F*_p_ in blueberry leaves under low temperature stress, whereas the application of Hypotaurine increased the reduction of *F*_p_.

**Figure 3:**
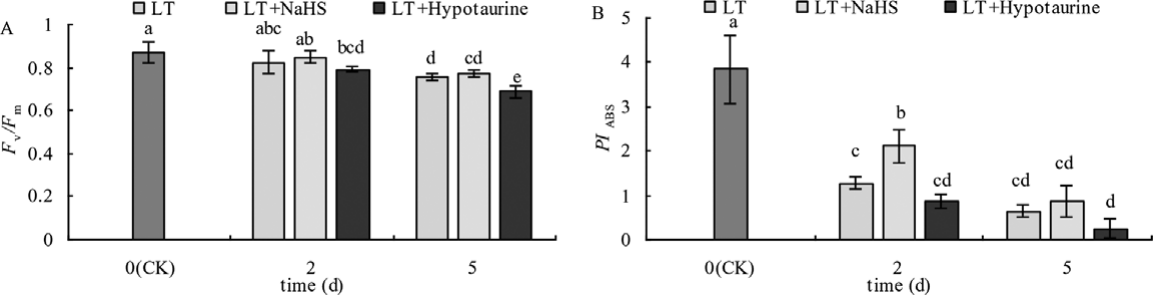
Effects of exogenous NaHS and Hypotaurine on *F*_v_/*F*_m_ **(A)** and *PI*_ABS_ **(B)** of blueberry leaves under low temperature stress. The data in the figure are from five replicated experiments (n = 5), and represent means ± standard error (SE). Different small letters show significant differences (*P* < 0.05).CK: room temperature control at 25°C; LT: low temperature treatment; LT + NaHS: low temperature treatment at 4–6°C after spraying 0.5 mmol·L^–1^ NaHS; LT + Hypotaurine: low temperature treatment at 4–6°C after spraying leaves with 200 μmol·L^–1^ hypotaurine.

With the increased duration of the low temperature treatment, *F*_v_/*F*_m_ and *PI*_ABS_ of blueberry leaves showed a decreasing trend. *PI*_ABS_ showed a greater decrease than *F*_v_/*F*_m_ (**Figure 3**). Under low temperature stress, there was no significant difference of *F*_v_/*F*_m_ between the LT+NaHS treatment and the LT treatment, but *PI*_ABS_ of blueberry leaves in the LT+NaHS treatment was higher than that in LT treatment by 65.35% (*P* < 0.05) and 36.51% (*P* > 0.05), respectively. Spraying with Hypotaurine resulted in an increase in the reduction of *F*_v_/*F*_m_ and *PI*_ABS_.

### Standardized O–P Curve and Standardized O–J Curve

**Figure 4:**
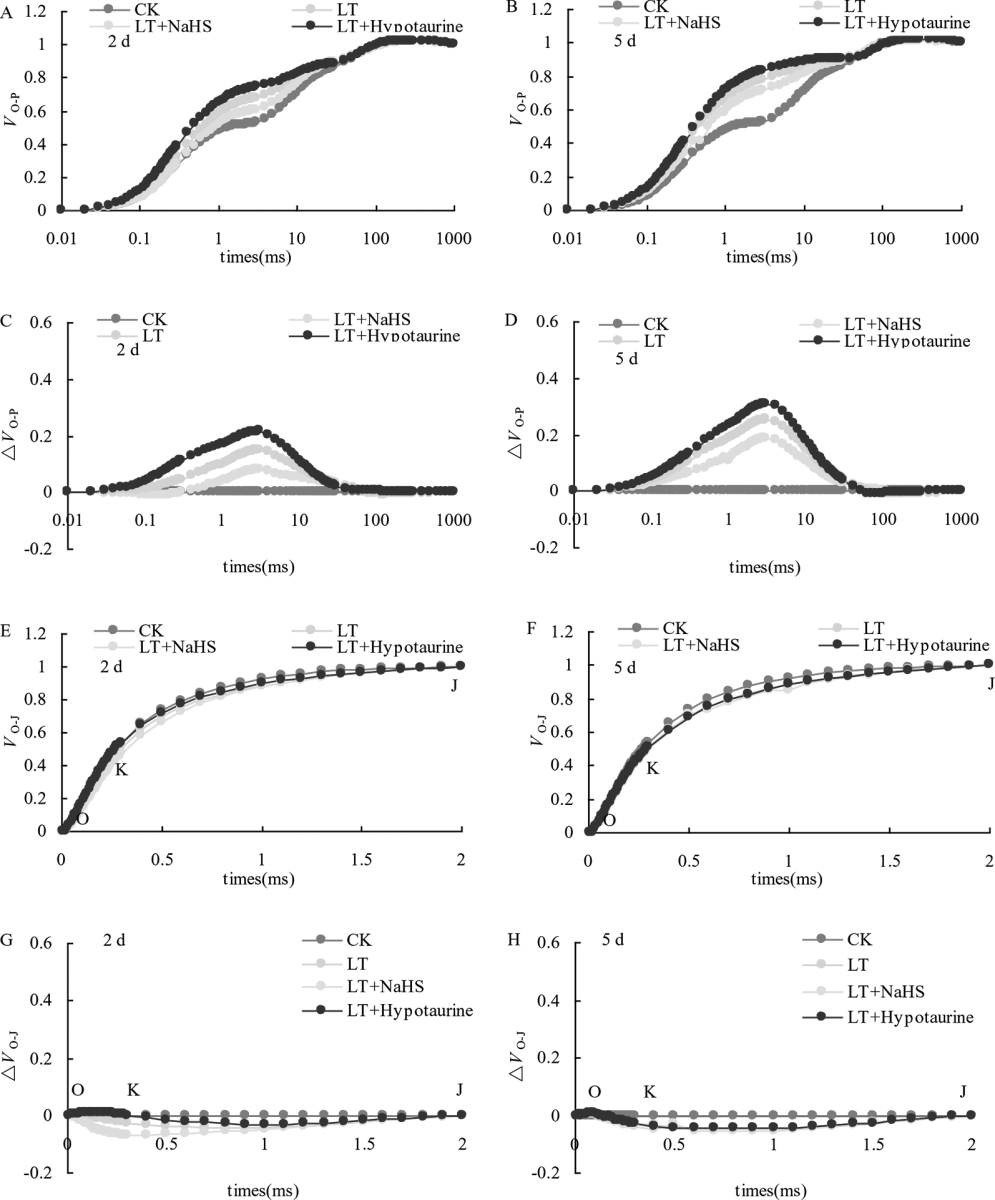
Effects of exogenous NaHS and Hypotaurine on *V*_O-P_ and *V*_O-J_ curves of blueberry leaves under low temperature stress. The data in the figure are from five replicated experiments (n = 5). CK: room temperature control at 25°C; LT: low temperature treatment; LT + NaHS: low temperature treatment at 4–6°C after spraying 0.5 mmol·L^–1^ NaHS; LT + Hypotaurine: low temperature treatment at 4–6°C after spraying leaves with 200 μmol·L^–1^ hypotaurine. Effects of exogenous NaHS and Hypotaurine on OJIP curves of blueberry leaves under low temperature stress. OJIP curves of blueberry leaves in different treatments were standardized by O-P (VO-P) **(A**, **B)**. The difference (ΔVO-P) between VO-P and CK **(C**, **D)**. OJIP curves were standardized by O-J (VO-J) **(E**, **F)**. The difference(ΔVO-J) between the VO-J curve and the CK **(G**, **H)**. The data in the figure are from five replicated experiments (n = 5). CK: room temperature control at 25°C; LT: low temperature treatment; LT + NaHS: low temperature treatment at 4–6°C after spraying 0.5 mmol•L-1 NaHS; LT + Hypotaurine: low temperature treatment at 4-6°C after spraying leaves with 200 μmol•L-1 hypotaurine.

**Figure 5:**
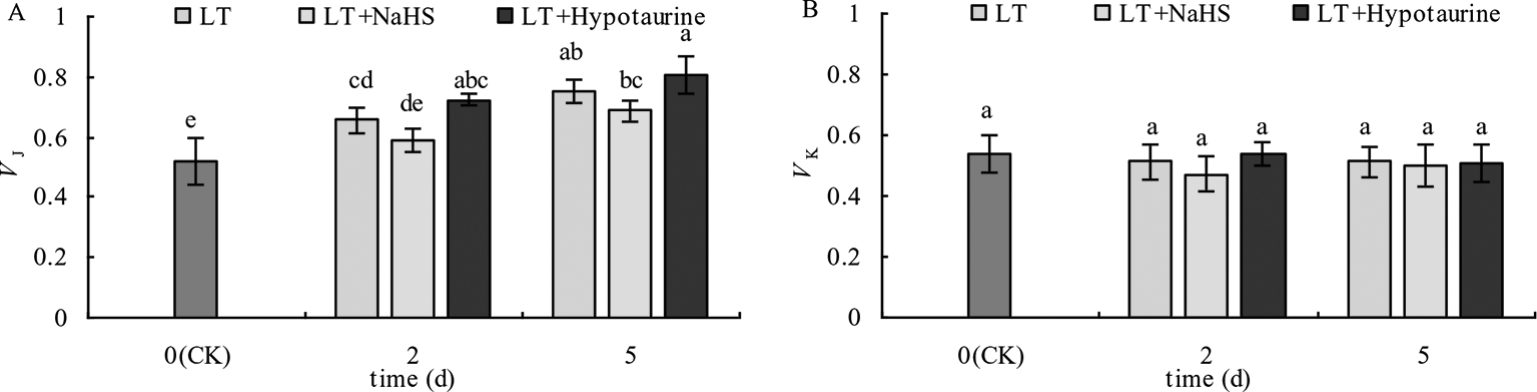
Effects of exogenous NaHS and Hypotaurine on *V*_J_ **(A)** and *V*_K_ **(B)** of blueberry leaves under low temperature stress. The data in the figure are from five replicated experiments (n = 5), and represent means ± standard error (SE). Different small letters show significant differences (*P* < 0.05). CK: room temperature control at 25°C; LT: low temperature treatment; LT + NaHS: low temperature treatment at 4–6°C after spraying 0.5 mmol·L^–1^ NaHS; LT + Hypotaurine: low temperature treatment at 4–6°C after spraying leaves with 200 μmol·L^–1^ hypotaurine.

OJIP curves of blueberry leaves in different treatments were standardized by O-P (*V*_O-P_) (**Figures 4A, B**). The difference (△*V*_O–P_) between *V*_O–P_ and CK (**Figures 4C, D**) showed that the relative variable fluorescence *V*_J_ at 2 ms of the *V*_O–P_ curve increased significantly under low temperature stress, and the increase was greater at the 5^th^ day than at the 2^nd^ day. Under low temperature stress, the *V*_J_ of blueberry leaves in the NaHS treatment was lower than that in LT treatment by 23.04% (*P* > 0.05) and 17.55% (*P* > 0.05) at the 2^nd^ and 5^th^ day, respectively, while the Hypotaurine treatment further increased *V*_J_ (**Figure 5A**).

OJIP curves were standardized by O–J (*V*_O–J_) (**Figures 4E, F**). The difference (△*V*_O–J_) between the *V*_O–J_ curve and the CK (**Figures 4G, H**) revealed that low temperature stress had little effect on *V*_K_ at 0.3 ms, and there was no significant difference between *V*_K_ and CK at the 2^nd^ and 5^th^ day of low temperature treatment. The effect of NaHS and Hypotaurine treatments on *V*_K_ was also not significant (**Figure 5B**).

### The Modulated Reflected Signal 820 nm (MR820 nm)

**Figure 6:**
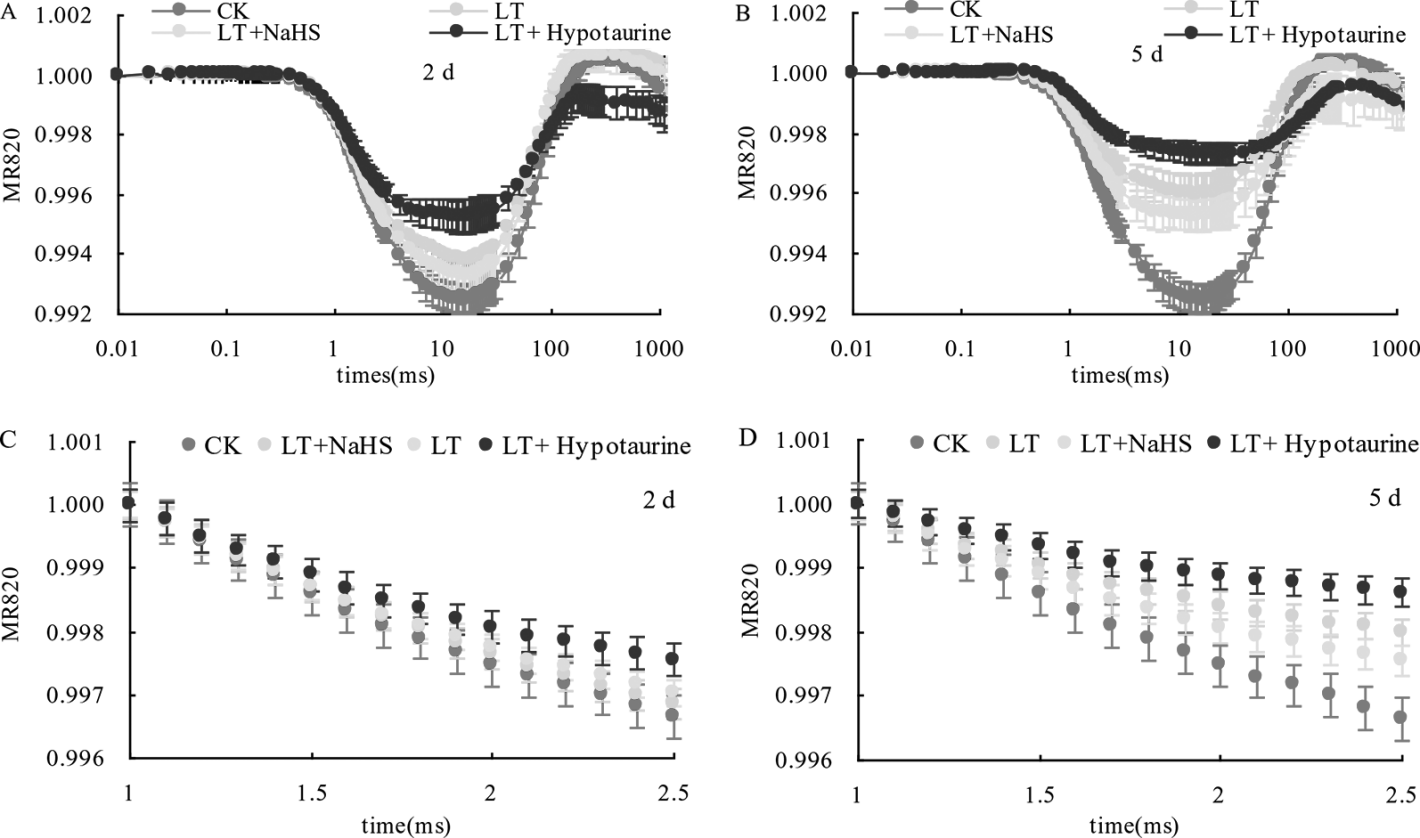
Effects of exogenous NaHS and Hypotaurine on the modulated reflected signal of 820 nm (MR820 nm) in blueberry leaves at the 2^nd^ **(A)** and 5^th^ **(B)** day of low temperature treatment and on the slope of the MR820 nm curve at the initial stage (1–2.5 ms) of decline at the 2^nd^ **(C)** and 5^th^ **(D)** day of treatment. The data in the figure are from five replicated experiments (n = 5). CK: room temperature control at 25°C; LT: low temperature treatment; LT + NaHS: low temperature treatment at 4–6°C after spraying 0.5 mmol·L^–1^ NaHS; LT + Hypotaurine: low temperature treatment at 4–6°C after spraying leaves with 200 μmol·L^–1^ hypotaurine.

**Figure 7:**
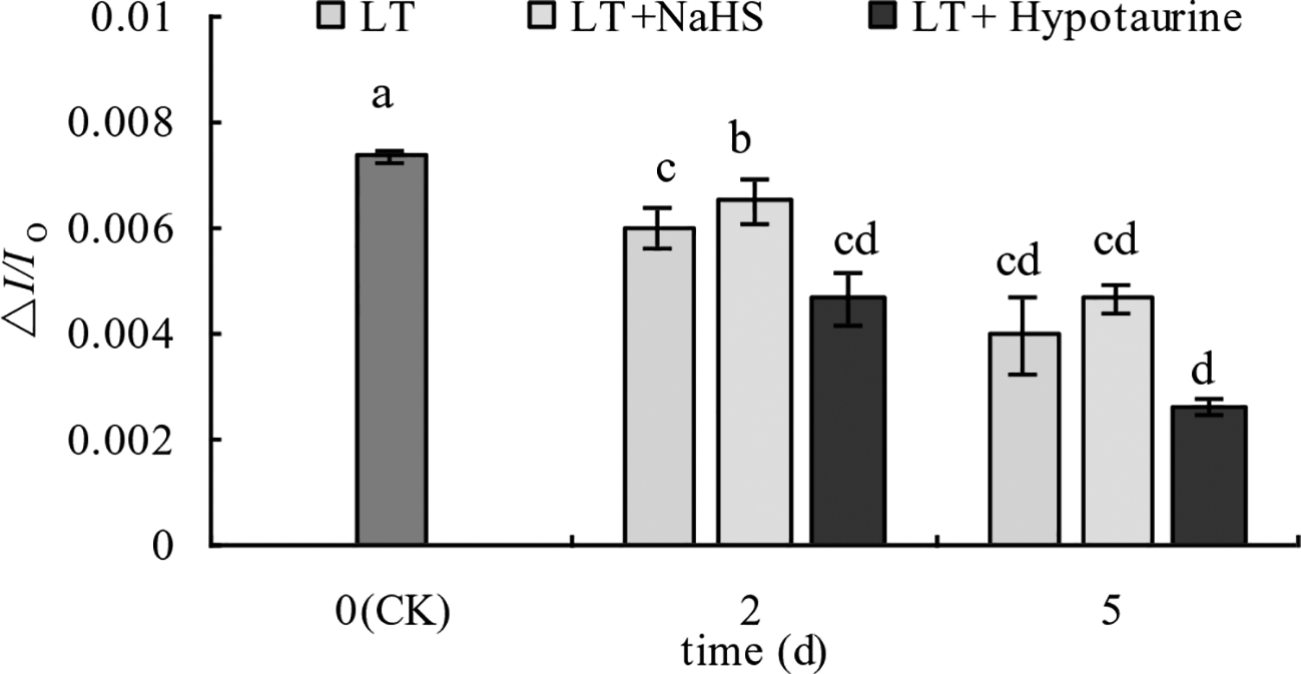
Effects of exogenous NaHS and Hypotaurine on △*I/I*_o_ of blueberry leaves under low temperature stress. The data in the figure are from five replicated experiments (n = 5), and represent means ± standard error (SE). Different small letters show significant differences (*P* < 0.05). CK: room temperature control at 25°C; LT: low temperature treatment; LT + NaHS: low temperature treatment at 4–6°C after spraying 0.5 mmol·L^–1^ NaHS; LT + Hypotaurine: low temperature treatment at 4–6°C after spraying leaves with 200 μmol·L^–1^ hypotaurine.

Under low temperature stress, the amplitude of the MR820 nm curve of blueberry leaves decreased (**Figures 6A, B**), and the slope of the MR820 nm curve at the initial stage (1–2.5 ms) decreased compared with the CK (**Figures 6C, D**). The decrease of the MR820 nm curve at the 5^th^ day of low temperature treatment was greater than that at the 2^nd^ day. Exogenous NaHS significantly alleviated the amplitude of the MR820 nm curve and minimized the decrease of the initial slope. In contrast, treatment with Hypotaurine showed the opposite effect. Quantitative analysis of △*I/I*_o_ changes (**Figure 7**) showed that △*I/I*_o_ of blueberry leaves decreased by 18.78% (*P* < 0.05) and 46.16% (*P* < 0.05) on the 2^nd^ and 5^th^ day of low temperature treatment, respectively. The decrease of △*I/I*_o_ in the LT + NaHS treatment was significantly lower than that in the LT treatment, whereas the Hypotaurine treatment maximized the decrease of △*I/I*_o_ under low temperature stress.

### Gas Exchange Parameters of Photosynthesis

**Figure 8:**
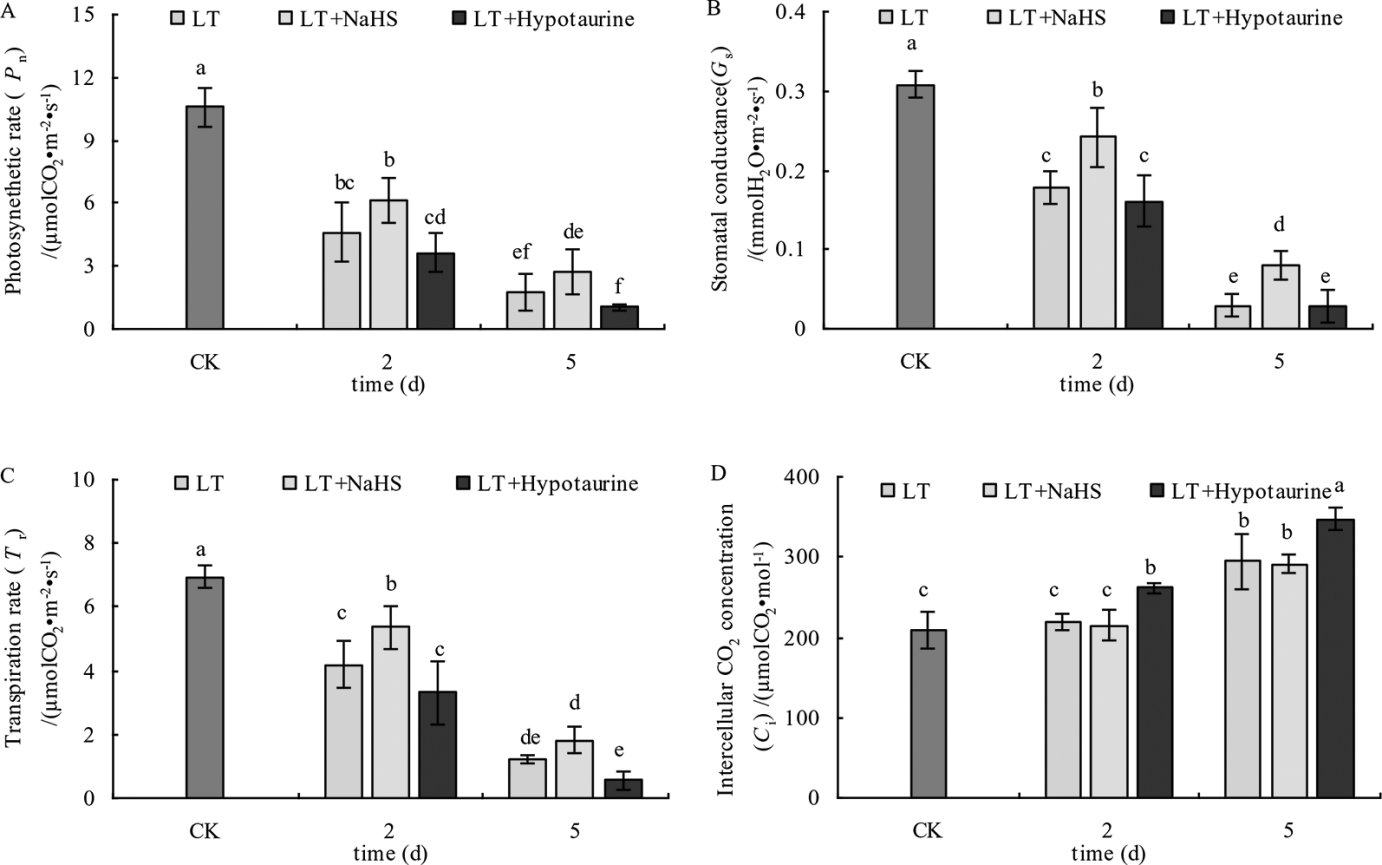
Effects of exogenous NaHS and Hypotaurine on net photosynthetic rate **(A)**, stomatal conductance **(B)**, transpiration rate **(C)**, and intercellular CO_2_ concentration **(D)** of blueberry leaves under low temperature stress. The data in the figure are from five replicated experiments (n=5), and represent means ± standard error (SE). Different small letters show significant differences (*P* < 0.05).CK: room temperature control at 25°C; LT: low temperature treatment; LT + NaHS: low temperature treatment at 4–6°C after spraying 0.5 mmol·L^–1^ NaHS; LT + Hypotaurine: low temperature treatment at 4–6°C after spraying leaves with 200 μmol·L^–1^ hypotaurine.

The results in **Figure 8** showed that the *P*_n_, *G*_s_, and *T*_r_ of blueberry leaves decreased significantly under low temperature stress; however, the decrease of *P*_n_, *G*_s_, and *T*_r_ was alleviated to varying degrees after spraying with exogenous NaHS. After spraying with Hypotaurine, *P*_n_, *G*_s_, and *T*_r_ showed a more evident decrease compared with the control. *C*_i_ in blueberry leaves did not change significantly at the 2^nd^ day of low temperature, but increased significantly at the 5^th^ day. Exogenous NaHS had no significant effect on *C*_i_ in blueberry leaves under low temperature stress, but Hypotaurine treatment increased *C*_i_ significantly.

### Change in the Activity of the Dark Reaction

**Figure 9:**
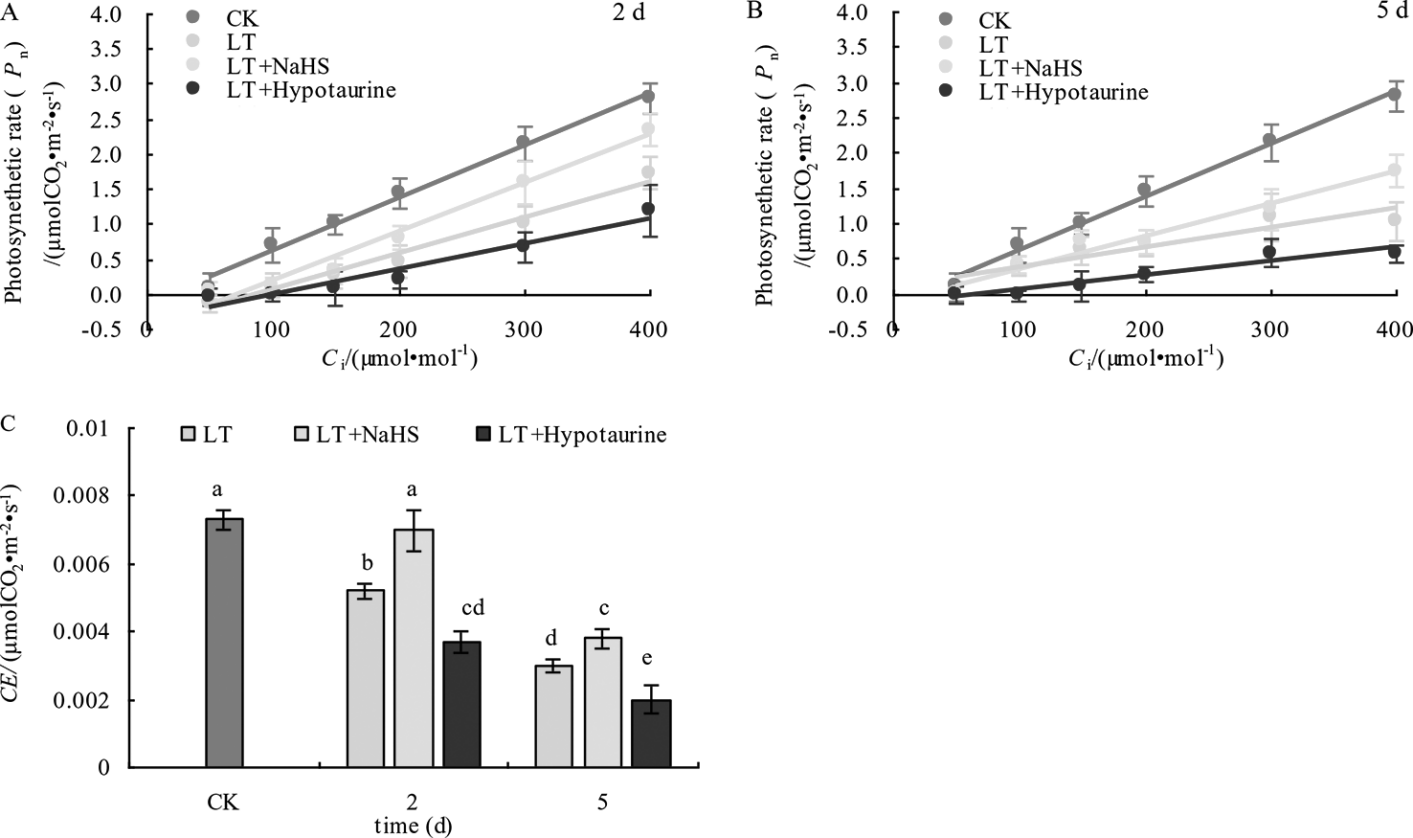
Effects of exogenous NaHS and HT on the initial slope **(A**, **B)** and *CE* **(C)** of the CO_2_ response curve of blueberry leaves under low temperature stress. The data in the figure are from five replicated experiments (n=5), and represent means ± standard error (SE). Different small letters show significant differences (*P* < 0.05).CK: room temperature control at 25°C; LT: low temperature treatment; LT + NaHS: low temperature treatment at 4–6°C after spraying 0.5 mmol·L^–1^ NaHS; LT + Hypotaurine: low temperature treatment at 4–6°C after spraying leaves with 200 μmol·L^–1^ hypotaurine.

The determination of the initial slope of the CO_2_ response curve (**Figures 9A, B**) and *CE* (**Figure 9C**) showed that *CE* in blueberry leaves decreased significantly under low temperature stress. However, *CE* in the LT + NaHS treatment was significantly greater than that in the LT treatment on the 2^nd^ and 5^th^ day of cold treatment (*P* < 0.05), whereas the decrease of *CE* in LT+ Hypotaurine treatment was significantly greater than that in LT treatment.

### Pro, H_2_O_2_, and MDA Content

**Figure 10:**
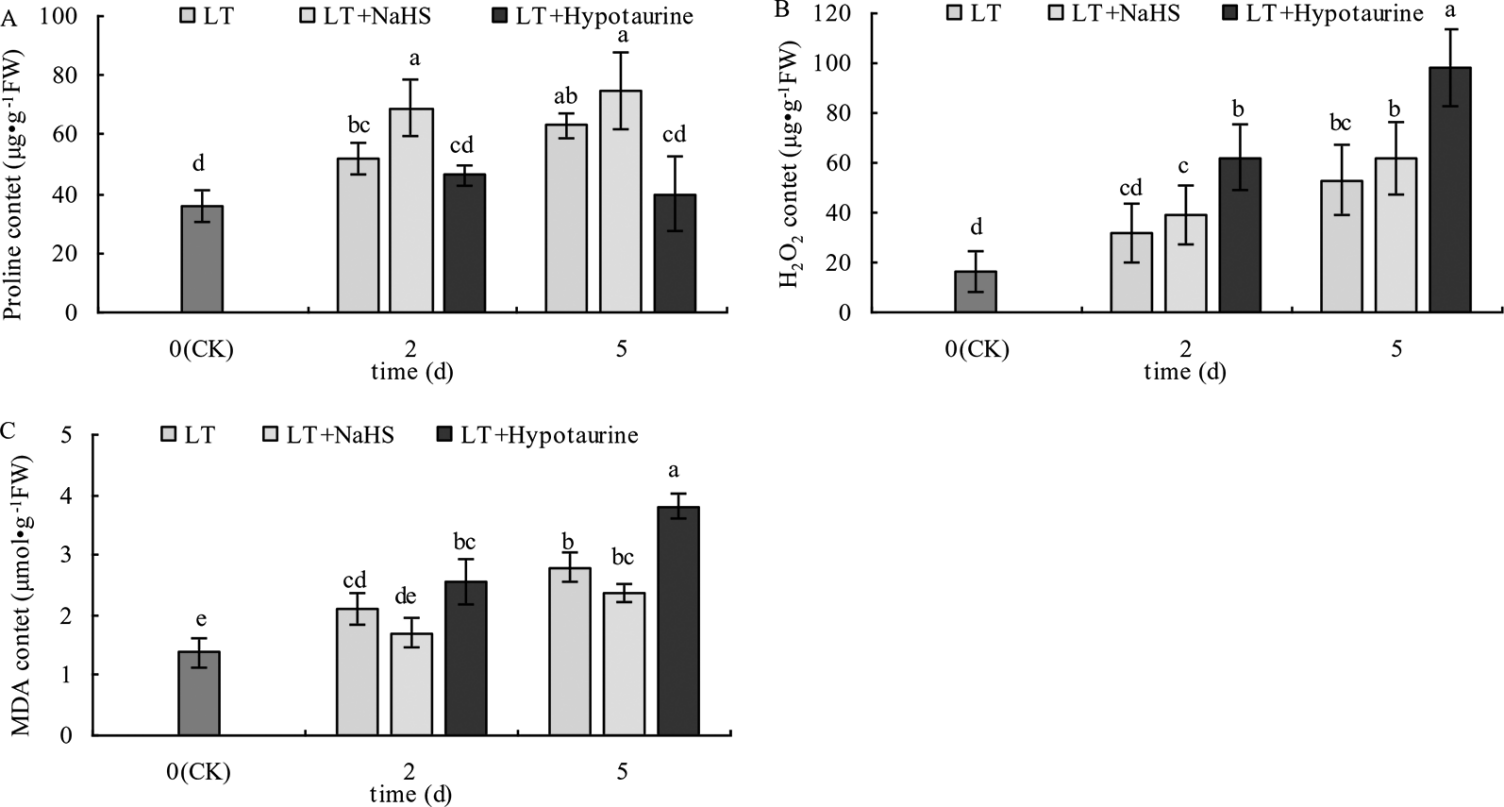
Effects of exogenous NaHS and Hypotaurine on proline (Pro) **(A)**, H_2_O_2_ **(B)**, and MDA **(C)** content in blueberry leaves under low temperature stress. The data in the figure are from three replicated experiments (n=3), and represent means ± standard error (SE). Different small letters show significant differences (*P* < 0.05). CK: room temperature control at 25°C; LT: low temperature treatment; LT + NaHS: low temperature treatment at 4–6°C after spraying 0.5 mmol·L^–1^ NaHS; LT + Hypotaurine: low temperature treatment at 4–6°C after spraying leaves with 200 μmol·L^–1^ hypotaurine.

With the prolongation of the low temperature treatment, the Pro, H_2_O_2_, and MDA content in blueberry leaves increased obviously (**Figure 10**). At the 2^nd^ and 5^th^ day of low temperature treatment, the Pro content of blueberry leaves treated with LT + NaHS increased by 32.69% (*P* < 0.05) and 19.05% (*P* > 0.05), respectively, compared with the plants treated with LT. The MDA content in the LT + NaHS treatment was 19.15% (*P* > 0.05) and 15.36% (*P* > 0.05) lower than that in LT treatment on the 2^nd^ and 5^th^ day of low temperature treatment, respectively. Therefore, spraying blueberry leaves with Hypotaurine significantly decreased Pro content and increased the accumulation of H_2_O_2_ and MDA content in blueberry leaves under low temperature stress.

## Discussion

Chloroplasts are the main site of plant photosynthesis and one of the organelles that is most sensitive to stress. The decrease of chlorophyll content in the chloroplast inhibits the absorption and utilization of light energy by plants (Zhang et al., 2016). In our study, the Chla, Chlb, and Chla+b content in blueberry leaves were significantly decreased under low temperature stress (**Figures 1A–C**), which indicated that low temperature stress could lead to chlorophyll degradation or inhibit chlorophyll synthesis. The addition of exogenous NaHS could promote chlorophyll synthesis or alleviate its degradation rate (Chen et al., 2011), and exogenous NaHS could also promote chlorophyll synthesis and chloroplast development in maize under iron deficient conditions (Chen et al., 2015). Our results are consistent with these reports. The treatment with exogenous NaHS prior to low temperature stress significantly alleviated the decrease of chlorophyll content. In contrast, the application of exogenous Hypotaurine increased the reduction of chlorophyll content, indicating that exogenous H_2_S could prevent the degradation of chlorophyll in blueberry leaves under low temperature stress. Carotenoids are involved in the absorption and transmission of light energy by plants, as well as have strong antioxidant capacity (Zhai et al., 2016), and beneficial to the photosystem II assembly and function (Zakar et al., 2016). In the carotenoid-reduced *Arabidopsis* szl1 mutant, the sensitivity of PSI and PSII to low temperature increased significantly (Cazzaniga et al., 2012). Low temperature stress induced the decrease of carotenoid content in blueberry leaves (**Figure 1D**) and exogenous H_2_S alleviated the degradation of Car in blueberry leaves under low temperature stress.

Low temperature stress often leads to the decrease of PSII activity in plants. The photoinhibition of PSII decreases linearly with the decrease of temperature in the range of 4 to 25°C (Sonoike, 2011). The relative fluorescence intensity at point P of the OJIP curve decreased significantly under low temperature stress, and *F*_v_/*F*_m_ and *PI*_ABS_ showed a decreasing trend, especially *PI*_ABS_ (**Figure 3**), indicating that low temperature led to the decrease of photochemical activity of PSII, and even photoinhibition. In addition, *V*_J_ increased significantly, whereas *V*_K_ did not change significantly. The increase of *V*_J_ reflects the inhibition of electron transfer from *Q*_A_ to *Q*_B_ on the PSII acceptor side (Zhang et al., 2017; Zhang et al., 2018a), while the increase of *V*_K_ is considered to be a specific marker of damage to oxygen-evolving complex on the PSII donor side (Zhang et al., 2018b). However, the change of *V*_K_ is not only affected by injury on the PSII donor side, but also by damage on PSII acceptor side. When the injury on the acceptor side is greater than that on the donor side, *V*_K_ does not increase significantly (Zhang et al., 2018c; Zhang et al., 2019b). Therefore, although the electron transfer from *Q*_A_ to *Q*_B_ on the PSII acceptor side of blueberry leaves was inhibited by low temperature, low temperature had little effect on the oxygen-evolving complex of the PSII donor side as *V*_K_ did not change. The inhibition of electron transport from *Q*_A_ to *Q*_B_ in the PSII acceptor side under stress conditions was mainly related to the degradation of D1 protein, while exogenous H2S can accelerate the turnover of D1 protein in wheat leaves under drought stress to improve the drought resistance of PSII function (Li et al., 2015). Therefore, exogenous NaHS significantly alleviated the increase of *V*_J_ under low temperature stress, while exogenous Hypotaurine increased *V*_J_, suggesting that exogenous H_2_S might protect the D1 protein in blueberry leaves under low temperature stress. Ultimately, this alleviates the photoinhibition of PSII.

In addition to the photoinhibition of PSII, PSI is also an important photoinhibition site under low temperature stress, especially in cold-sensitive plants. Low temperature stress makes PSI more prone to photoinhibition than PSII, and its degree of photoinhibition is often greater than PSII, and more challenging to recover (Terashima et al., 1994; Sonoike and Terashima, 1994; Zhang et al., 2010). We found that the PSI activity of blueberry leaves decreased under low temperature stress. The decrease of PSI activity was significantly alleviated by the exogenous application NaHS, while PSI activity was further decreased by Hypotaurine, an H_2_S scavenger, under low temperature stress. These data indicated that exogenous H_2_S could increase the PSI activity under low temperature stress. A previous study has reported that the photoinhibition of PSI is mainly related to the increase of reactive oxygen species in PSI (Sonoike, 1996). Thus, the accumulation of H_2_O_2_ under low temperature stress caused by Hypotaurine (**Figure 10B**) is an important reason for the increase of PSI photoinhibition.

Some studies have reported that exogenous H_2_S improved the drought resistance of *Arabidopsis thaliana* by inducing stomatal closure (Jin et al., 2017), which was mainly due to the reduction of the stomatal diameter caused by H_2_S (Jin et al., 2011) or the increase in the expression of the mitogen activated protein kinase gene to prevent over opening of stomata under low temperature stress (Du et al., 2017). However, other studies have shown that exogenous H_2_S improved photosynthetic capacity of rice leaves by increasing stomatal aperture and density, and NaHS induced stomatal opening in *Arabidopsis thaliana* by inhibiting NO production (Lisjak et al., 2010). Therefore, the function of H_2_S in plant stomatal movement is still controversial and requires further study. The *G*_s_ of blueberry leaves decreased rapidly under low temperature stress, leading to the decrease of *T*_r_ and *P*_n_.

After spraying leaves with exogenous NaHS, the decrease of *G*_s_ and *T*_r_ was significantly lower than that of the control. Moreover, the decrease of *P*_n_ was also alleviated to varying degrees with the application of NaHS, whereas spraying with Hypotaurine aggravated stomatal closure and further decreased the photosynthetic rate under low temperature stress (**Figure 8**). This indicates that exogenous H_2_S could improve photosynthetic capacity of blueberry leaves under low temperature stress by promoting stomatal opening. Although the *G*_s_ in the exogenous NaHS treatment was significantly higher than that in non-sprayed NaHS treatment, the variation between the *P*_n_ was not significant. These results indicated that the application of exogenous H_2_S also increased photosynthetic capacity under low temperature stress *via* non-stomatal factors. Non-stomatal factors, such as the decrease of photosynthetic enzyme activity under stress, are also important factors that limit plant photosynthesis. Under severe stress, non-stomatal factors often play a major role in limiting plant photosynthesis (Zhang et al., 2019b). At the 5^th^ day of low temperature stress, the *C*_i_ increased significantly (**Figure 8D**), indicating that the reason for the decrease of photosynthetic capacity caused by long-term (5 d) low temperature stress was due to the limitation of non-stomatal factors (Arena and Vitale, 2018; Zhang et al., 2019a) Exogenous H_2_S can promote the transport of CO_2_ (Espie et al., 1989), the expression of photosynthesis-related enzymes, and the redox modification of thiol groups to improve photosynthesis Application of exogenous H_2_S can also promote the protein and gene expression of Ribulose-1,5-Bisphosphate Carboxylase (Rubisco) and phenol pyruvate carboxylase in maize leaves under iron deficiency conditions (Chen et al., 2011). In our study, spraying NaHS increased the *CE* under low temperature stress, while spraying Hypotaurine decreased *CE* (**Figure 9**). Therefore, the reason why exogenous H_2_S can increase photosynthetic capacity of blueberry leaves under low temperature stress is not only related to the increase of induced stomatal conductance, but also possibly related to the fact that exogenous H_2_S is beneficial to CO_2_ fixation in dark reaction under low temperature stress, which may be related to the protection of dark reaction-related enzymes.

H_2_S also interacts with other hormones and signaling substances in plants, such as NO (Chen et al., 2011; Wang et al., 2012), SA (Amooaghaie et al., 2017), and Ca^2+^ (Li et al., 2012; Qiao et al., 2015). In addition, as a very important cell signaling molecule, H_2_O_2_ content is regulated by H_2_S in many physiological processes during plant growth and development (Fang H. et al., 2014; Zhang et al., 2008). H_2_S can be used as an upstream signaling molecule of H_2_O_2_ to promote the seed germination as described for mung bean (Li et al., 2012). Li et al. found that H_2_S could improve salt tolerance in *Arabidopsis thaliana* roots, and this process required the active participation of H_2_O_2_ (Li et al., 2015). With the increased duration of low temperature stress, H_2_O_2_ content in blueberry leaves increased significantly, although exogenous NaHS treatment promoted the increase of H_2_O_2_ content in blueberry leaves to some extent, the difference was not significant, which may be related to the increase of antioxidant enzyme activity or accumulation of antioxidants by NaHS under low temperature stress. Exogenous Hypotaurine significantly increased H_2_O_2_ content under low temperature stress. A low concentration of H_2_O_2_ can be used as a signaling substance in plant response to stress, and a high concentration of H_2_O_2_ can cause oxidative damage to plant cells (Li et al., 2014). Therefore, exogenous Hypotaurine aggravates oxidative damage under low temperature stress.

H_2_S can alleviate the membrane peroxidation under low temperature stress by regulating the activity of antioxidant enzymes in hawthorn fruits (Cheng et al., 2016), mitigate the oxidative damage caused by Al by increasing the antioxidant capacity of wheat (Aghdam et al., 2018), and reduce the MDA content by enhancing the activity of antioxidant enzymes in alfalfa seedlings under Cd stress (Zhang et al., 2010). In addition, H_2_S can also induce the accumulation of ascorbic acid and glutathione in plants to improve its antioxidant capacity (Cui et al., 2014). Under low temperature stress, the increase of MDA content of blueberry leaves was significantly alleviated in NaHS treatment, while the membrane peroxidation of leaves was intensified in Hypotaurine treatment, which was consistent with the change of H_2_O_2_ content (**Figure 10B**).

**Figure 11:**
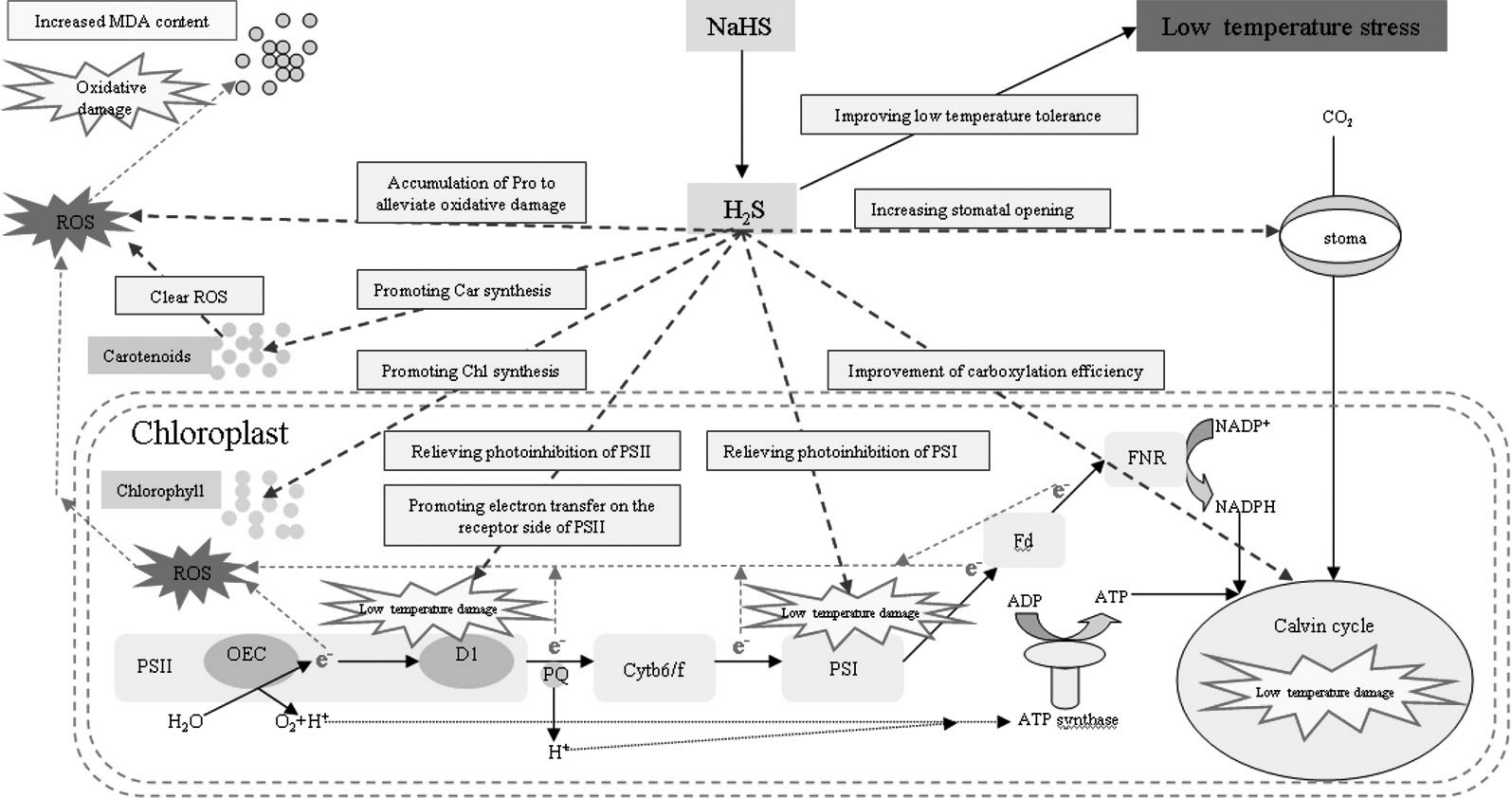
Mechanisms of exogenous H_2_S donor (NaHS) in alleviating photosynthesis inhibition under low temperature stress.

Under stress, plant cells actively accumulate small molecules to regulate their osmotic potential and maintain their normal water content (Shan et al., 2011). Exogenous H_2_S could control the water potential and relative water content of spinach leaves by regulating the synthesis of soluble sugar, polyamine, and glycine betaine to enhance the adaptability of spinach seedlings to drought (Kaur and Asthir, 2015). The accumulation of Pro plays an important role in improving stress resistance of plants (Chen et al., 2016). Pro is also an inducer of osmotic stress-related genes and is a scavenger of reactive oxygen species (Hong et al., 2000; Theocharis et al., 2012), which plays an important role in improving the stability of plant cell membranes under stress (Lu and Becker, 2015). Luo et al. (2015) has found that exogenous H2S could increase the Pro content of banana under low temperature stress and significantly enhances their cold tolerance, which was mainly related to H_2_S increasing the activity of 1-pyrroline-5-carboxylate synthetase and decreasing the activity of proline dehydrogenase (Mansour, 2013). Li et al. (2015) also demonstrated that exogenous H_2_S improved the heat tolerance of maize, which was related to the Pro accumulation induced by exogenous H_2_S. The results of the present experiment are consistent with these previous reports. Under low temperature stress, the accumulation of Pro leaves increased, and spraying with exogenous NaHS promoted the Pro accumulation, while spraying with Hypotaurine had the opposite effect (**Figure 10A**). Therefore, the accumulation of Pro is an adaptive mechanism to low temperature stress, and the accumulation of Pro induced by exogenous H_2_S plays an active role in improving its low temperature tolerance. The mechanism of exogenous H_2_S donor (NaHS) alleviating photosynthesis inhibition under low temperature stress is summarized in **Figure 11**.

## Conclusions

NaHS, an exogenous H_2_S donor, significantly alleviated the degradation of chlorophyll and carotenoids in blueberry leaves under low temperature stress. NaHS also increased the activities of PSII and PSI, of which the electron transfer from *Q*_A_ to *Q*_B_ on the acceptor side of PSII may be the site of primary activity of H2S. Exogenous H_2_S also promoted stomatal opening and photosynthetic carbon assimilation ability under low temperature stress. Promoting the accumulation of Pro plays an important role in improving the low temperature tolerance of blueberry by exogenous H_2_S. In contrast, spraying blueberry leaves with Hypotaurine, an H_2_S scavenger, aggravated the photoinhibition and oxidative damage of blueberry leaves. In conclusion, the application of exogenous H_2_S improved the tolerance of blueberry to low temperature stress, which was mainly related to the improvement of photosynthetic capacity and the accumulation of Pro in blueberry leaves.

## Data Availability Statement

All datasets generated for this study are included in the article/supplementary material.

## Author Contributions

XT, BA and XL conceived and designed experiments; All the authors performed the experiments and analyzed the data; XT and BA wrote the manuscript and prepared the figures and/or tables. XT, BA and XL reviewed drafts of the paper. XT and BA contributed equally to this work.

## Funding

This work was supported by National key Research and Development Program of China (2017YFC0504205); Science and Technology Development Program of Jilin Province in China (2016041201XH).

## Conflict of Interest

The authors declare that the research was conducted in the absence of any commercial or financial relationships that could be construed as a potential conflict of interest.

## References

[B1] Aghdam M. S. Mahmoudi R. Razavi F. Rabiei V. Soleimani A. (2018). Hydrogen sulfide treatment confers chilling tolerance in hawthorn fruitduring cold storage by triggering endogenous H2S accumulation, enhancingantioxidant enzymes activity and promoting phenols accumulation. Sci. Hort. 238, 264–271. 10.1016/j.scienta.2018.04.063

[B2] Alexieva V. Sergiev I. Mapelli S. Karanov E. (2001). The effect of drought and ultraviolet radiation on growth and stress markers in pea and wheat. Plant Cell Environ. 24 (12), 1337–1344. 10.1046/j.1365-3040.2001.00778.x

[B3] Amooaghaie R. Zangenemadar F. Enteshari S. (2017). Role of two-sided crosstalk between NO and H_2_S on improvement of mineral homeostasis and antioxidative defense in Sesamum indicum under lead stress. Ecotoxicol. Environ. Saf. 139, 210–218. 10.1016/j.ecoenv.2017.01.037 28142110

[B4] Arena C. Vitale L. (2018). Chilling-induced reduction of photosynthesis is mitigated by exposure to elevated CO_2_ concentrations. Photosynthetica 56, 1259–1267. 10.1007/s11099-018-0843-3

[B5] Bates L. S. Waldren R. P. Teare I. D. (1973). Rapid determination of free proline for water stress studies. Plant Soil 39, 205–207. 10.1007/BF00018060

[B6] Cazzaniga S. Li Z. Niyogi K. K. Bassi R. Dall′Osto L. (2012). The *Arabidopsis* szl1 mutant reveals a critical role of -carotene in photosystem I photoprotection. Plant Physiol. 159 (4), 1745–1758. 10.1104/pp.112.201137 23029671 PMC3425210

[B7] Chen J. Wu F. H. Wang W. H. Wang W. H. Hu W. J. Simon M. (2011). Hydrogen sulphide enhances photosynthesis through promoting chloroplast biogenesis, photosynthetic enzyme expression, and thiol redox modification in Spinacia oleracea seedlings. J. Exp. Bot. 62 (13), 4481–4493. 10.1093/jxb/err145 21624977 PMC3170546

[B8] Chen J. Wang W. H. Wu F. H. Chun Y. Y. Ting W. L. Xue J. D. (2013). Hydrogen sulfide alleviates aluminum toxicity in barley seedlings. Plant Soil 362 (1-2), 301–318. 10.1007/s11104-012-1275-7

[B9] Chen J. Wu F. H. Shang Y. T. Wang W. H. Hu W. J. Simon M. (2015). Hydrogen sulphide improves adaptation of Zea mays seedlings to iron deficiency. J. Exp. Bot. 66 (21), 6605–6622. 10.1093/jxb/erv368 26208645 PMC4623679

[B10] Chen J. Shang Y. T. Wang W. H. Chen X. Y. He E. M. Zheng H. L. (2016). Hydrogen sulfide-mediated polyamines and sugar changes are involved in hydrogen sulfide-induced drought tolerance in *Spinacia oleracea* seedlings. Front. Plant Sci. 7, 1173. 10.3389/fpls.2016.01173 27540388 PMC4972840

[B11] Cheng W. Zhang L. Jiao C. J. Su M. Yang T. Zhou L. N. (2013). Hydrogen sulfide alleviates hypoxia-induced root tip death in *Pisum sativum*. Plant Physiol. Biochem. 70 (1), 278–286. 10.1016/j.plaphy.2013.05.042 23800663

[B12] Cheng D. D. Lui M. J. Sun X. B. Zhao M. Chow W. S. Sun G. Y. (2016). Light suppresses bacterial population through the accumulation of hydrogen peroxide in tobacco leaves infected with Pseudomonas syringae pv. tabaci. Front. Plant Sci. 7, 512. 10.3389/fpls.2016.00512 27148334 PMC4838606

[B13] Christou A. (2013). Hydrogen sulfide induces systemic tolerance to salinity and non-ionic osmotic stress in strawberry plants through modification of reactive species biosynthesis and transcriptional regulation of multiple defence pathways. J. Exp. Bot. 64 (7), 1953–1966. 10.1093/jxb/ert055 23567865 PMC3638822

[B14] Coyne P. Bingham G. (1978). Photosynthesis and stomatal light responses in snap beans exposed to hydrogen sulfide and ozone. Air Repair 128 (11), 1119–1123. 10.1080/00022470.1978.10470715

[B15] Cui W. Chen H. Zhu K. Jin Q. Xie Y. Cui J. (2014). Cadmium-induced hydrogen sulfide synthesis is involved in cadmium tolerance in *Medicago sativa* by reestablishment of reduced (Homo) glutathione and reactive oxygen species homeostases. PloS One 9 (10), e109669. 10.1371/journal.pone.0109669 25275379 PMC4183592

[B16] Du X. Z. Jin Z. P. Liu D. M. Yang G. D. Pei Y. X. (2017). Hydrogen sulfide alleviates the cold stress through MPK4 in Arabidopsis thaliana. Plant Physiol. Biochem. 120, 112–119. 10.1016/j.plaphy.2017.09.028 29024849

[B17] Duan B. B. Ma Y. H. Jiang M. G. Yang F. Ni L. Lu W. (2015). Improvement of photosynthesis in rice (*Oryza sativa* L.) as a result of an increase in stomatal aperture and density by exogenous hydrogen sulfide treatment. Plant Growth Regul. 75 (1), 33–44. 10.1007/s10725-014-9929-5

[B18] Espie G. S. Miller A. G. Canvin D. T. (1989). Selective and reversible inhibition of active CO2 transport by hydrogen sulfide in a Cyanobacterium. Plant Physiol. 91 (1), 387–394. 10.1104/pp.91.1.387 16667030 PMC1062004

[B19] Fang T. Cao Z. Li J. Shen W. Huang L. (2014). Auxin-induced hydrogen sulfide generation is involved in lateral root formation in tomato. Plant Physiol. Biochem. 76 (5), 44–51. 10.1016/j.plaphy.2013.12.024 24463534

[B20] Fang H. H. Jing T. Liu Z. Q. Zhang L. P. Jin Z. P. Pei Y. X. (2014). Hydrogen sulfide interacts with calcium signaling to enhance the chromium tolerance in *Setaria italica*. Cell Calcium 56 (6), 472–481. 10.1016/j.ceca.2014.10.004 25459298

[B21] Fu L. H. Hu K. D. Hu L. Y. Li Y. H. Hu L. B. Yan H. (2014). An antifungal role of hydrogen sulfide on the postharvest pathogens *Aspergillus niger* and *Penicillium italicum*. PloS One 9, e104206. 10.1371/journal.pone.0104206 25101960 PMC4125178

[B22] Hancock J. T. Whiteman M. (2016). Hydrogen sulfide signaling: interactions with nitric oxide and reactive oxygen species. Ann. New York Acad. Sci. 1365 (1), 5–14. 10.1111/nyas.12733 25782612

[B23] Hong Z. Lakkineni K. Zhang Z. Verma D. P. (2000). Removal of feedback inhibition of delta(1)-pyrroline-5-carboxylate synthetase results in increased proline accumulation and protection of plants from osmotic stress. Plant Physiol. 122 (4), 1129–1136. 10.1104/pp.122.4.1129 10759508 PMC58947

[B24] Hu K. D. Wang Q. Hu L. Y. Gao S. P. Wu J. Li Y. H. (2014). Hydrogen sulfide prolongs postharvest storage of fresh-cut pears (*Pyrus pyrifolia*) by alleviation of oxidative damage and inhibition of fungal growth. PloS One 9, e85524. 10.1371/journal.pone.0085524 24454881 PMC3893216

[B25] Hua Z. Hao J. Jiang C. X. Wang S. H. Wei Z. J. Luo J. P. (2010). Hydrogen sulfide protects soybean seedlings against drought-induced oxidative stress. Acta Physiol. Plantarum 32 (5), 849–857. 10.1007/s11738-010-0469-y

[B26] Jia H. Hu Y. Fan T. Li J. (2015). Hydrogen sulfide modulates actin-dependent auxin transport *via* regulating ABPs results in changing of root development in *Arabidopsis*. Sci. Rep. 5, 8251. 10.1038/srep08251 25652660 PMC4317700

[B27] Jin Z. P. Shen J. J. Qiao Z. J. Yang G. D. Wang R. Pei T. X. (2011). Hydrogen sulfide improves drought resistance in *Arabidopsis thaliana*. Biochem. Biophys. Res. Commun. 414 (3), 481–486. 10.1016/j.bbrc.2011.09.090 21986537

[B28] Jin Z. P. Xue S. W. Luo Y. N. Tian B. H. Fang H. H. Li H. (2013). Hydrogen sulfide interacting with abscisic acid in stomatal regulation responses to drought stress in *Arabidopsis*. Plant Physiol. Biochem. 62 (1), 41–46. 10.1016/j.plaphy.2012.10.017 23178483

[B29] Jin Z. P. Wang Z. Q. Ma Q. X. Sun L. M. Zhang L. P. Liu Z. Q. (2017). Hydrogen sulfide mediates ion fluxes inducing stomatal closure in response to drought stress in Arabidopsis thaliana. Plant Soil 419 (1-2), 141–152. 10.1007/s11104-017-3335-5

[B30] Jin Z. P. Sun L. M. Yang G. D. Pei Y. X. (2018). Hydrogen sulfide regulates energy production to delay leaf senescence induced by drought stress in *Arabidopsis*. Front. Plant Sci. 9, 1722. 10.3389/fpls.2018.01722 30532763 PMC6265512

[B31] Joanna T. D. Anna B. Jan F. Piotr Z. Emilia J. Marlena R. (2019). Global analysis of gene expression in maize leaves treated with low temperature: I. Moderate chilling (14°C). Plant Sci. 177 (6), 648–658. 10.1016/j.plantsci.2009.09.001

[B32] Kaur G. Asthir B. (2015). Proline: a key player in plant abiotic stress tolerance. Biol. Plantarum 59 (4), 609–619. 10.1007/s10535-015-0549-3

[B33] Lai D. Mao Y. Zhou H. Li F. Wu M. Zhang J. (2014). Endogenous hydrogen sulfide enhances salt tolerance by coupling the reestablishment of redox homeostasis and preventing salt-induced K^+^ loss in seedlings of *Medicago sativa*. Plant Sci. 225 (8), 117–129. 10.1016/j.plantsci.2014.06.006 25017167

[B34] Li Z. G. He Q. Q. (2015). Hydrogen peroxide might be a downstream signal molecule of hydrogen sulfide in seed germination of mung bean (*Vigna radiata*). Biologia 70 (6), 753–759. 10.1515/biolog-2015-0083

[B35] Li Z. G. Gong M. Xie H. Tan L. Li J. (2012). Hydrogen sulfide donor sodium hydrosulfide-induced heat tolerance in tobacco (*Nicotiana tabacum* L.) suspension cultured cells and involvement of Ca^2+^and calmodulin. Plant Sci. 185–186 (4), 185–189. 10.1016/j.plantsci.2011.10.006 22325880

[B36] Li J. S. Jia H. L. Wang J. Cao Q. H. Wen Z. C. (2014). Hydrogen sulfide is involved in maintaining ion homeostasis *via* regulating plasma membrane Na^+^/H^+^ antiporter system in the hydrogen peroxide-dependent manner in salt-stress *Arabidopsis thaliana* root. Protoplasma 251 (4), 899–912. 10.1007/s00709-013-0592-x 24318675

[B37] Li H. Gao M. Q. Xue R. L. Wang D. Zhao H. J. (2015). Effect of hydrogen sulfide on D1 protein in wheat under drought stress. Acta Physiol. Plantarum 37 (11), 225. 10.1007/s11738-015-1975-8

[B38] Li Z. G. Min X. Zhou Z. H. (2016). Hydrogen sulfide: a signal molecule in plant cross-adaptation. Front. Plant Sci. 7, 1621. 10.3389/fpls.2016.01621 27833636 PMC5080339

[B39] Lichtenthaler H. K. (1987). Chlorophylls and carotenoids: pigments of photosynthetic biomembranes. Method Enzymol. 148, 350–382. 10.1016/0076-6879(87)48036-1

[B40] Lisjak M. Srivastava N. Teklic T. Civale L. Lewandowski K. Wilson I. (2010). A novel hydrogen sulfide donor causes stomatal opening and reduces nitric oxide accumulation. Plant Physiol. Biochem. 48 (12), 931–935. 10.1016/j.plaphy.2010.09.016 20970349

[B41] Lisjak M. Teklic T. Wilson I. D. Wood M. Whiteman M. Hancock J. (2011). Hydrogen sulfide effects on stomatal apertures. Plant Signaling Behav. 6 (10), 1444–1446. 10.4161/psb.6.10.17104 PMC3256366 21904118

[B42] Liu R. Lal R. (2015). Effects of low-level aqueous hydrogen sulfide and other sulfur species on lettuce (*Lactuca sativa*) seed germination. Commun. Soil Sci. Plant Anal. 46 (5), 576–587. 10.1080/00103624.2014.998341

[B43] Lu Z. Becker D. F. (2015). Connecting proline metabolism and signaling pathways in plant senescence. Front. Plant Sci. 6, 522. 26347750 10.3389/fpls.2015.00552 PMC4544304

[B44] Luo Z. Li D. Du R. X. Mou W. X. (2015). Hydrogen sulfide alleviates chilling injury of banana fruit by enhanced antioxidant system and proline content. Scientia Hortic. 183 (183), 144–151. 10.1016/j.scienta.2014.12.021

[B45] Mansour M. M. F. (2013). Plasma membrane permeability as an indicator of salt tolerance in plants. Biol. Plantarum 57 (1), 1–10. 10.1007/s10535-012-0144-9

[B46] Mustafa A. K. Gadalla M. M. Sen N. Kim S. Mu W. Gazi S. K. (2009). H_2_S signals through protein S-sulfhydration. Sci. Signal. 2 (96), 72. 10.1126/scisignal.2000464 PMC2998899 19903941

[B47] Qiao Z. Tao J. Liu Z. Q. Zhang L. P. Jin Z. P. Liu D. M. (2015). H_2_S acting as a downstream signaling molecule of SA regulates Cd tolerance in *Arabidopsis*. Plant Soil 393 (1-2), 137–146. 10.1007/s11104-015-2475-8

[B48] Scuffi D. Álvarez C. Laspina N. Gotor C. Lamattina L. García-Mata C. (2014). Hydrogen sulfide generated by L-cysteine desulfhydrase acts upstream of nitric oxide to modulate abscisic acid-dependent stomatal closure. Plant Physiol. 166 (4), 2065–2076. 10.1104/pp.114.245373 25266633 PMC4256879

[B49] Shan C. J. Zhang S. L. Li D. F. Zhao Y. Z. Tian X. L. Zhao X. L. (2011). Effects of exogenous hydrogen sulfide on the ascorbate and glutathione metabolism in wheat seedlings leaves under water stress. Acta Physiol. Plantarum 33 (6), 2533–2540. 10.1007/s11738-011-0746-4

[B50] Shen J. R. Terashima I. Katoh S. (1990). Cause for dark, chilling-induced inactivation of photosynthetic oxygen evolving system in cucumber leaves. Plant Physiol. 93, 1354–1357. 10.1104/pp.93.4.1354 16667624 PMC1062679

[B51] Shi H. Ye T. Han N. Bian H. Liu X. Chan Z. (2015). Hydrogen sulfide regulates abiotic stress tolerance and biotic stress resistance in *Arabidopsis*. J. Integr. Plant Biol. 57 (7), 628–640. 10.1111/jipb.12302 25329496

[B52] Sonoike K. Terashima I. (1994). Mechanism of photosystem-I photoinhibition in leaves of *Cucumis sativus* L. Planta 194 (2), 287–293. 10.1007/BF00196400

[B53] Sonoike K. (1996). Degradation of psaB gene product, the reaction center subunit of photosystem I, is caused during photoinhibition of photosystem I: possible involvement of active oxygen species. Plant Sci. 115 (2), 157–164. 10.1016/0168-9452(96)04341-5

[B54] Sonoike K. (2011). Photoinhibition of photosystem I. Physiol. Plantarum 142, 56–64. 10.1111/j.1399-3054.2010.01437.x 21128947

[B55] Strasser R. J. Srivastava A. Govindjee (1995). Polyphasic chlorophyll a fluorescence transient in plants and cyanobacteria. Photochem. Photobiol. 61 (1), 32–42. 10.1111/j.1751-1097.1995.tb09240.x

[B56] Strauss A. J. Kruger G. H. Strasser R. J. Van Heerden P. D. (2010). The role of low soil temperature in the inhibition of growth and PSII function during dark chilling in soybean genotypes of contrasting tolerance. Physiol. Plantarum 131 (1), 89–105. 10.1111/j.1399-3054.2007.00930.x 18251928

[B57] Terashima I. Funayama S. Sonoike K. (1994). The site of photoinhibition in leaves of *Cucumis sativus* L.at low temperatures is photosystem I, not photosystem II. Planta 193 (2), 300–306. 10.1007/BF00192544

[B58] Theocharis A. Clément C. Barka E. A. (2012). Physiological and molecular changes in plants grown at low temperatures. Planta 235 (6), 1091–1105. 10.1007/s00425-012-1641-y 22526498

[B59] Wang X. Chen S. Zhang H. Shi L. Cao F. Guo L. (2010). Desiccation tolerance mechanism in resurrection fern-ally Selaginella tamariscina revealed by physiological and proteomic analysis. J. Proteome Res. 9, 6561–6577. 10.1021/pr100767k 20923197

[B60] Wang Y. Li L. Cui W. Xu S. Shen W. Wang R. (2012). Hydrogen sulfide enhances alfalfa (*Medicago sativa*) tolerance against salinity during seed germination by nitric oxide pathway. Plant Soil 351, 107–119. 10.1007/s11104-011-0936-2

[B61] Zakar T. Laczko-Dobos H. Toth T. N. Gombos Z. (2016). Carotenoids assist in cyanobacterial photosystem II assembly and function. Front. Plant Sci. 7, 295. 10.3389/fpls.2016.00295 27014318 PMC4785236

[B62] Zhai S. Xia X. He Z. (2016). Carotenoids in staple cereals: metabolism, regulation, and genetic manipulation. Front. Plant Sci. 7, 227. 10.3389/fpls.2016.01197 27559339 PMC4978713

[B63] Zhang H. Hu L. Y. Hu K. D. He Y. D. Wang S. H. Luo J. P. (2008). Hydrogen sulfide promotes wheat seed germination and alleviates oxidative damage against copper stress. J. Integr. Plant Biol. 50 (12), 1518–1529. 10.1111/j.1744-7909.2008.00769.x 19093970

[B64] Zhang H. Wang M. J. Hu L. Y. Wang S. H. Hu K. D. Bao L. J. (2010a). Hydrogen sulfide promotes wheat seed germination under osmotic stress. Russian J. Plant Physiol. 57 (4), 532–539. 10.1134/S1021443710040114

[B65] Zhang Z. S. Jia Y. J. Gao H. Y. Zhang L. T. Li H. D. Meng Q. W. (2010). Characterization of PSI recovery after chilling-induced photoinhibition in cucumber (*Cucumis sativus* L.) leaves. Planta 234 (5), 883–889. 10.1007/s00425-011-1447-3 21647604

[B66] Zhang H. Tan Z. Q. Hu L. Y. Wang S. H. Luo J. F. Jones R. L. (2010b). Hydrogen sulfide alleviates aluminum toxicity in germinating wheat seedlings. J. Integr. Plant Biol. 52, 556–567. 10.1111/j.1744-7909.2010.00946.x 20590986

[B67] Zhang H. H. Zhong H. X. Wang J. F. Sui X. Xu N. (2016). Adaptive changes in chlorophyll content and photosynthetic features to low light in *Physocarpus amurensis Maxim* and *Physocarpus opulifolius* "Diabolo". Peer J. 4 (3), e 2125. 10.7717/peerj.2125 PMC4924129 27366639

[B68] Zhang H. H. Xu N. Li X. Jin W. W. Tian Q. Sun G. Y. (2017). Overexpression of 2-Cys Prx increased salt tolerance of photosystemII in tobacco. Int. J. Agric. Biol. 19 (4), 735–745. 10.17957/IJAB/15.0348

[B69] Zhang H. H. Xu N. Wu X. Y. Wang J. R. Ma S. L. Li X. (2018a). Effects of 4 kinds of sodium salt stress on plant growth, PS II and PS I function in leaves of Sorghum. J. Plant Interact. 13 (1), 506–513. 10.1080/17429145.2018.1526978

[B70] Zhang H. H. Xu N. Teng Z. Y. Wang J. R. Ma S. L. Wu X. Y. (2019a). 2-Cys Prx plays a critical role in scavenging H_2_O_2_ and protecting photosynthetic function in leaves of tobacco seedlings under drought stress. J. Plant Interact. ,14 (1), 119–128. 10.1080/17429145.2018.1562111

[B71] Zhang H. H. Feng P. Yang W. Sui X. Li X. Zhang R. T. (2018b). Effects of flooding stress on the photosynthetic apparatus of leaves of two Physocarpus cultivars. J. For. Res. 39 (4), 1049–1059. 10.1007/s11676-017-0496-2

[B72] Zhang H. H. Li X. Zhang S. B. Yin Z. P. Zhu W. X. Li J. B. (2018c). Rootstock alleviates salt stress in grafted mulberry seedlings: physiological and PSII Function responses. Front. Plant Sci. 9, 1806. 10.3389/fpls.2018.01806 30619391 PMC6297837

[B73] Zhang H. H. Shi G. L. Shao J. Y. Li X. Li M. B. Meng L. (2019b). Photochemistry and proteomics of mulberry (*Morus alba* L.) seedlings under NaCl and NaHCO_3_ stress. Ecotoxicol. Environ. Safety 184 (30), 109624. 10.1016/j.ecoenv.2019.109624 31487570

[B74] Zhang H. Tang J. Liu X. P. Wang Y. Yu W. Peng W. Y. (2019c). Hydrogen sulfide promotes root Organogenesis in ipomoea batatas, salix matsudana and glycine max. J. Integr. Plant Biol. 51 (12), 1086–1094. 10.1111/j.1744-7909.2009.00885.x 20021556

